# Experiences of Boosting Inpatient Exercise After HipFracture Surgery Using An Alternative Workforce - A Qualitative Study

**DOI:** 10.1186/s12877-024-04756-1

**Published:** 2024-02-23

**Authors:** Benny Lau, Marie K. March, Alison R. Harmer, Sarah Caruana, Christopher Mahony, Sarah Dennis

**Affiliations:** 1 Sydney School of Health Sciences, Faculty of Medicine and Health, University of Sydney, Camperdown, Australia; 2 Physiotherapy Department, Fairfield Hospital, South Western Sydney Local Health District, Warwick Farm, Australia; 3 Physiotherapy Department, Blacktown Mt Druitt Hospitals, Western Sydney Local Health District, Blacktown, Australia; 4 Ingham Institute of Applied Medical Research, Liverpool, Australia; 5 Physiotherapy Department, Hornsby Ku-ring-gai Hospitals, Northern Sydney Local Health District, Hornsby, Australia

**Keywords:** Hip fracture, Physiotherapy, Implementation, Experiences, Allied health assistant, Student

## Abstract

**Background:**

Thrice-daily physiotherapy immediately following surgical repair of hip fracture has been shown to be safe and to reduce total hospital length of stay. However, implementing this is challenging with respect to health service funding and staffing. A novel approach may be to utilize an alternative workforce (allied health staff and student physiotherapists) to deliver two of the three daily treatments. However, how patients and staff may view such an approach is unknown. Thus, the aim of this qualitative study was to explore the views of inpatients with surgical repair of a hip fracture, their carers, health care professionals, and physiotherapy students about the implementation and acceptability of thrice-daily physiotherapy, with two sessions delivered by the alternative workforce (the BOOST study).

**Methods:**

Semi-structured interviews and focus groups with patients, carers, health professionals and physiotherapy students. All interviews were digitally recorded and transcribed via verbatim. The transcripts were coded, and the data analysed via inductive thematic analysis.

**Results:**

A total of 37 interviews (32 one-to-one interviews and five focus group interviews) were analysed. Five main themes were identified: (1) individual perceptions of the intervention: inpatients/carer/staff/student, (2) implementation within the service and organisational context, (3) implementation strategies that were effective, (4) improvements to implementation strategies/barriers to implementation/unsuccessful strategies and (5) future directions of BOOST.

**Conclusions:**

The qualitative data revealed that higher frequency physiotherapy was well-received by inpatients and that staff/students involved in providing care perceived it as a safe, acceptable and valuable practice. Implementation of higher daily frequency of physiotherapy using an alternative workforce may feasibly be adopted for inpatients following hip fracture surgery.

**Trial registration:**

This study was approved by the Human Research Ethics Committee (HREC) of the Western Sydney Local Health District (2020/ETH02718). Mutual recognition of approval was subsequently obtained from Northern Sydney Local Health District HREC.

## Background

The average age of people admitted to hospital in 2019 following a hip fracture was 82 years in both Australia and New Zealand, with people aged above 90 years comprising 25% of this cohort [1]. This older population is at risk of frailty, with numerous risk factors that may contribute to subsequent hip fractures, morbidity, and mortality. Hip fracture is a debilitating injury that may arise from a variety of traumatic experiences, including high and low trauma fractures [2, 3]. The number of hip fractures worldwide is predicted to increase by about four-fold from an overall incidence estimated between 1.25 and 1.66 million in 1990 to an estimated 4.5 to 6.5 million by 2050 [4]. Hip fractures impose a huge financial encumbrance on the Australian healthcare system due to the need for surgery and hospitalisation [3, 5]. In New South Wales (NSW), Australia, there is a projected rise in annual acute hip fracture care costs from AUD$163.9 million in 2011 to between AUD$224.8 to $345.0 million in 2036 [6]. Therefore, it is highly important to participate in physical rehabilitation during the acute phase of recovery after hip fracture surgery to improve patients’ general health and overall function [7, 8].

Best practice physiotherapy encourages commencing mobility within 24 h after surgery [9, 10]. A randomised controlled trial (RCT) found that patients who first mobilised on postoperative days 1 or 2 (early ambulation) significantly increased walking distance and reduced assistance required for mobility at one-week post-surgery compared to those who first mobilised on postoperative days 3 or 4 [11]. Patients engaged in early mobilisation were more likely to be discharged directly home from acute care and less likely to need high-level care [11]. Conventionally, mobilisation occurs with a physiotherapist once per day after surgical repair of hip fracture [9, 10]. Another RCT investigated the effects of thrice-daily mobilisation after surgical repair of hip fracture, with two daily physiotherapy sessions delivered by a physiotherapist and an additional session delivered by an allied health assistant [12]. This was safe and significantly reduced total (combined acute and rehabilitation) hospital length of stay [12]. Despite this high-quality evidence, as yet there is no evidence supporting successful implementation in a typical clinical setting, and hence no knowledge of the acceptability of such an approach to inpatients and clinicians [12].

The aim of our embedded qualitative study was to examine, in the acute period after surgical repair of hip fracture, the experiences and acceptability of higher frequency physiotherapy implemented by an alternative workforce (allied health staff and physiotherapy students) among inpatients, carers, and relevant healthcare workers.

## Methods

### Study design

This paper reports the qualitative aspects of a prospective implementation study of higher frequency physiotherapy in the acute period after surgical repair of hip fracture [13]. Thrice-daily exercise therapy sessions were implemented for ten weeks in two public hospitals from the Western Sydney Local Health District and Northern Sydney Local Health District, NSW, Australia. The BOOST intervention included one session provided daily by a physiotherapist, which involved mobilisation (e.g. transfers, walking, and progression to negotiating stairs) and any other exercises that the physiotherapist deemed appropriate. Two additional exercise therapy sessions were then implemented by the alternative workforce (either allied health assistants or physiotherapy students) trained in the delivery of the BOOST protocol. In these sessions, sit-to-stand exercise was prescribed as the core exercise for all patients, with dose prescribed by the treating physiotherapist. However, chair-based, bed-based, and upper limb exercises were provided if patients did not consent to sit-to-stand practice, but still wanted to participate. Patients were given the opportunity for three physiotherapist-prescribed exercise sessions per day. Experiences of the BOOST protocol from inpatients (and/or their carers), alternative workforce staff involved in therapy implementation, physiotherapy staff, orthogeriatric clinical staff and managers were obtained via semi-structured interviews or focus groups. The perspectives were explored using inductive thematic analysis, conducted by two authors.

### Inclusion criteria and sample size

Inpatients in Blacktown Mount Druitt Hospital or Hornsby Ku-ring-Gai Hospital with surgical repair of a hip fracture were included in the broader study. All patients and relevant staff were approached regarding participation in the qualitative aspects of the study. Patients were given the option to defer to carers to provide an interview if desired. *A priori* we anticipated there would be 12 eligible inpatient participants per month per site based on previous inpatient data. As many staff as possible who were involved and identified as key stakeholders in the implementation process were purposefully sampled. The only occupational group not approached for interview was the orthopaedic surgical team due to a lack of availability. Written informed consent was obtained from all participants who volunteered to provide qualitative data.

### Data collection

The data collection for this qualitative study was obtained via semi-structured interviews with inpatients and/or their nominated carers, and semi-structured interviews or focus groups with physiotherapy staff (consisting of NSW Health Level 4 Acute Clinical Educators, Level 3 Orthopaedic Senior Physiotherapists, and Level 1/2 rotational physiotherapists, all of whom had a clinical caseload), orthogeriatric clinical staff (i.e. geriatricians, nursing and an occupational therapist), relevant health service managers, and the alternative workforce which included pre-registration physiotherapy students and allied health assistants. Physiotherapy staff provide care to patients through assessment, planning, implementation and evaluation of interventions which include but are not limited to mobilisation. Geriatricians are medical officers who specialise in the care of elderly patients. Nursing staff collaborate with the multidisciplinary team to monitor patients’ conditions and assess their needs to provide the best possible care and advice, observing and interpreting patients’ symptoms, and devising individualised care plans. Occupational therapists help an individual who has an injury, illness or a disability which affects their ability to undertake tasks of everyday life such as eating, showering, shopping and going to work. Health service managers are responsible for the strategic, financial and day-to-day management of their relevant team within the hospital (e.g. physiotherapy, nursing etc.). Pre-registration students are still enrolled in their physiotherapy courses at university and participated in the study whilst on a 5-week clinical placement at the hospitals where we conducted the study. The number of clinical placements undertaken prior to participation in our study varied between none and four according to the university program and the year of enrolment at university. The students were either undertaking an undergraduate (entry-level) or postgraduate (Masters-level; i.e. requiring completion of a previous qualifying course, e.g. exercise science) physiotherapy course via various different universities, and thus, knowledge and experience differed to some degree between students. Allied health assistants provide assistance and support to the team in the delivery of allied health services to patients/clients of the hospital, under the supervision of an allied health professional.

An interview guide was created for our study using the Consolidated Framework for Implementation Research (CFIR) and used for all interviewees [14]. The interviews explored interviewees’ experiences and perceptions of the BOOST intervention. Interviews were conducted by three local site-based clinicians who were study investigators, but who did not provide treatment to inpatients or have direct clinical supervision of staff or students participating in the implementation of the BOOST intervention. Inpatients and/or their nominated carers were interviewed towards the end of their acute stay or shortly after their discharge from the acute ward. Interviews were conducted face-to-face, except for one which was done via telephone according to the preference of the interviewee. Interviews were conducted one-to-one where possible however, to facilitate participation, focus groups were available for staff members. Staff were interviewed in private rooms after the end of the implementation phase. Where possible, inpatients and carers were invited to a private room, however this was often not possible due to the severe functional limitations of inpatients. Carers often preferred to stay at the bedside of their loved one during interview. We did not encounter any particular concerns regarding interview participation for patients despite the interviews being completed whilst they were still receiving care (i.e. prior to being discharged). Every patient who was included in our intervention was offered an interview, and/or, if they preferred, the opportunity to defer to or consult with their carer. In order to minimise participant burden, member checking was not conducted; however, participants were given the opportunity to provide information after their interview as desired, and to provide feedback at the end of their interview. Inpatient and carer interviews were brief and ranged from four to 15 min, while staff interviews ranged from 30 to 60 min. Interviews were recorded digitally. De-identified recordings were transcribed via verbatim by an independent transcription service.

### Data analysis

The de-identified transcripts were read and re-read by two authors and initial codes and themes independently generated through line-by-line coding via the six-phase approach to thematic analysis suggested by Braun and Clarke [15]. The two authors then discussed codes and themes to reach agreement. To provide further triangulation, detailed discussion regarding coding and identification of themes was undertaken with another two authors to enhance trustworthiness of the study [16]. An inductive approach to thematic analysis was performed using NVivo [17]. Direct quotations from interviewees were used to support the identified themes.

## Results

In total, 32 one-to-one interviews and five focus group interviews were conducted to obtain qualitative data from 51 interviewees. There were 25 eligible inpatients (16 from Blacktown Mount Druitt Hospital and 9 from Hornsby Ku-ring-Gai Hospital). Of those 25 patients, one had a total hip replacement, eight had a hemiarthroplasty, three had dynamic hip screws, 11 had short intramedullary nails and two had long intramedullary nails. 12 inpatients and one nominated carer consented to participate in the interviews. The 12 interviewed inpatients had an average age of 81.2 years (SD = 7.7), and 64% (*n* = 7) of them were female. All staff members who were involved in the study at both sites consented to participate in the interviews (with the exception of orthopaedic surgeons as the orthopaedic teams at both sites were unavailable due to workforce demands). Interviewed staff comprised 12 physiotherapists (three were interviewed individually; nine were interviewed in three focus groups), 16 pre-registration physiotherapy students (eight were interviewed individually; eight were interviewed in one focus group), an allied health assistant, four health service managers (three were individual interviews, one health service manager was in a focus group with a clinician), and five orthogeriatric clinical staff (four were individual interviews, one clinician was in a focus group with a health service manager).

Five main themes were identified that described the perspectives of inpatients or their nominated carers, physiotherapists, the alternative workforce, orthogeriatric clinical staff, and relevant service managers on utilising an alternative workforce to administer two additional physiotherapy exercise sessions per day for patients after surgery for hip fracture. These were: (1) individual perceptions of the intervention: inpatients/carer/staff/student, (2) implementation within the service and organisational context, (3) implementation strategies that were effective, (4) improvements to implementation strategies/barriers to implementation/unsuccessful strategies and (5) future directions of BOOST.

### 1. Individual perceptions of the intervention: inpatients/carer/staff/student

The additional exercise sessions were perceived by inpatients/carer, staff and students as aligning with patient goals. The exercises chosen as well as the increased therapy time were viewed as an effective evidence-based approach for inpatients to remain active and provide the necessary benefits of mobility and strengthening during hospitalisation.

*“You’re not sitting around going stale…keep moving” - Patient 1*.

*“Less time sitting around getting stiff in between.”– Orthogeriatric Clinical Staff 1*.

The higher daily frequency of physiotherapy was also viewed by the alternative workforce as a practice that was basic and easy to implement.

*“…exercises were really simple, they were easy to teach, they were easy for the patient to follow. It was easy to do at the bedside as well…” - Physiotherapy Student 4*.

The dosage of extra physiotherapy was perceived as suitable by many of the inpatients.

*“No, it wasn’t too much…it was probably just the right amount.”- Patient 9*.

*“…whilst they may have been not overly receptive or enthusiastic about the actual project, [the inpatients] still participated and there [were] no complaints to my knowledge.” - Physiotherapist 5*.

Inpatients felt that the physiotherapists and physiotherapy students were able to build rapport with them, which made the inpatients feel safe and supported. Most importantly, using an alternative workforce was not perceived to increase risk to the inpatients, and the orthogeriatric clinical staff were quite happy about having students who were adequately trained and supervised.

*“I don’t think there’s any particular medical risk to it…”– Orthogeriatric Clinical Staff 1*.

Pain was for the most part adequately managed throughout the implementation of the BOOST intervention via effective multidisciplinary team communication and coordination.

*“[the nursing staff] said to me a couple of times if you want the stuff [pain medication] or if you’ve got pain, you tell us. So yeah, there has been no hassles.”- Patient 2*.

However, poor pain management was an issue for some inpatients/a carer as it was perceived to have a negative consequence on the effort provided by patients.

*“she’s feeling a little bit of pain but then goes, oh, no-no, that’s too sore, I can’t do it… they’re remembering from the day before, I’ve had to do this three times, it was painful. Where if it’s a gradual thing over a couple of days…”– Patient Carer 1*.

### 2. Implementation within the service and organisational context

Clinicians perceived patients with hip fracture as a priority treatment group for their respective hospital services and found that BOOST aligned well with allied health strategy plans by integrating research into practice. The higher frequency model of care also assisted with exceeding current standards of care.

Standard practice was quite variable in terms of discussing with patients the frequency of physiotherapy sessions, as the focus tended to be on encouraging participation when the opportunity arose. There were no formal parts of the study or interview process that told the included patients that “usual care” was once daily. Individual staff or students may have mentioned this ad hoc but we did not specify what staff could discuss, or not discuss. Despite this, some inpatients perceived that more therapy than usual was beneficial to their recovery, thus highlighting that they viewed BOOST as a helpful means of getting them more functionally mobile sooner.

*Interviewer: “…do you think exercising three times a day was good for you?”… “…[exercising three times a day] might help us to get out of the bed. I especially - think I want to fast get out of the bed. I don’t want to lie down more time on that bed.” - Patient 2*.

*“Having that known exercise routine also makes it very familiar and also helps the patient see their own growth and track their progress.” - Physiotherapy Student 3*.

By facilitating the development of independence, BOOST could potentially assist nursing staff by enabling inpatients to assist with nursing tasks. This is because the inpatients would be more functionally capable and independent to perform simple tasks such as mobilising and activities of daily living.

*“I think it benefits certainly the nursing staff because the patients are going to be more compliant with getting up out of the chair themselves.”– Physiotherapy Student 4*.

*“Because having it three times a day was sort of– in the morning when they were getting up to go to the toilet or for personal care, and then again in the afternoon if they, say, wanted to return back to bed after sitting out in a chair for most of the morning, and then again in the afternoon. Definitely.”– Orthogeriatric Clinical Staff 1*.

BOOST was perceived by the alternative workforce, physiotherapists, clinicians and managers to accelerate inpatients’ recovery process, which had subsequent benefits for the hospital such as reducing hospital costs and increasing bed availability.

*“I think it gave them the opportunity to get up and start walking and therefore hopefully reducing their length of stay in in-patient rehab.” - Orthogeriatric Clinical Staff 2*.

*“I think [BOOST benefitted the hospital] because if the goal is for them to discharge earlier then I see that as being a benefit, freeing up the beds quicker.– Physiotherapy Student 4*.

*“…probably because of the extra therapy, [patients] probably could have ended up going straight home”– Health Service Manager / Orthogeriatric Clinical Staff Focus Group*.

Since most elderly inpatients with hip fracture were discharged to further rehabilitation, BOOST seemed to prepare these inpatients well for their next destination.

*“So, we were able to progress them quicker, get them to home potentially quicker, and also get them to rehab in a better functional state than we normally send patients to rehab…” - Physiotherapist 4*.

Physiotherapy students participating as part of the alternative workforce reflected that participation in BOOST was a novel opportunity to participate in research, and to improve skills in communication and rapport-building with the inpatients.

*“I also feel like my skills with communication and with motivating patients was improved a lot with the specialisation.” - Physiotherapy Student 2*.

*“I think it’s a good educational opportunity for students to kind of put their head into research a little bit” - Physiotherapist 7*.

Using an alternative workforce was also viewed as beneficial for physiotherapy student educators to help them cater for a variety of student abilities. Students who were more capable and independent were allocated to deliver BOOST treatments (often on another hospital ward), allowing the clinical educator more time to attend to those students who required more supervision and education.

### 3. Implementation strategies that were effective

Physiotherapists and the alternative workforce felt that the orientation process prior to delivery of the BOOST study was comprehensive, and they felt as though their roles and the aims of BOOST were clear.

*“It was explained quite well in terms of who we’re going to be dealing with, what we’re doing and why we’re doing it.” - Physiotherapy Student 3*.

The wide variety of exercises allowed individualisation of treatment and modification for inpatients who might not be as adherent on the day for reasons such as being in pain or feeling fatigued.

*“…there was enough scope to tailor it…there was the bed exercises, but also sitting out in the chair exercises as well and you could just kind of pick your own adventure in that way.” - Allied Health Assistant 1*.

In addition, the timing and scheduling of treatment sessions did not seem to trouble the inpatients as staff implementing BOOST demonstrated flexibility. The alternative workforce were confident in being able to provide the additional treatment sessions for the inpatients.

*“…there was a couple of times they actually came and then rescheduled, they went and saw someone else because Mum was still eating. Yeah, so no, that certainly never disrupted anything.” - Patient Carer 1*.

*“I [could] see this patient on time, twice a day and I have the time for it.”– Physiotherapy Student 3*.

### 4. Improvements to implementation strategies/barriers to implementation/unsuccessful strategies

Suggestions were made by physiotherapists as well as the alternative workforce to make the BOOST orientation program more practical and possibly include a ward tour, practical training, and demonstrations.

*“The orientation gave me a bit of like a theory background, just like in my mind, but it wasn’t until I had to apply it that I fully understood it.” - Physiotherapy Student 1*.

*“Maybe adding - because you know how you do the scenarios - maybe adding a scenario is, what to do if you find the patient in bed as well.”– Physiotherapy Focus Group 3*.

Physiotherapy staff believed that implementing additional sessions of physiotherapy may be difficult with a larger caseload of inpatients or a lack of certainty with inpatient lists, although the latter could be mitigated by providing physiotherapists with a list of upcoming inpatients eligible for BOOST earlier in the day.

*“…when the number of patients surpassed the number of educators, that’s when it became quite problematic, I found.” - Physiotherapist 5*.

*“… it would be nice to know prior to 10 o’clock or prior to the day whether or not a BOOST patient is coming up on the list.” - Physiotherapy Focus Group 2*.

Although most interviewees believed that the dosage of extra physiotherapy was suitable, one inpatient and one staff member thought that the implementation of additional sessions of exercises may be too much for some inpatients.

*“For the people who are sick it’s a bit too much. If you’re all right, not feeling very sore, it’s okay, because he try feeling more better. You see? That’s it.”– Patient 3*.

*“Some patients might not want to participate three times a day.” - Orthogeriatric Clinical Staff 1*.

The implementation of BOOST also led to some unintended consequences, such as the view expressed below about how the additional sessions of physiotherapy exercise could potentially interfere with other allied health disciplines’ interventions.

*“So, I’d say yes in the fact that they got their extra sessions. I say no in the fact that I’ve been really busy and then the patients have been unavailable so I haven’t been involved with them as much as I normally would because you go and try to see a patient and they’re having their second session with the physio. So then - and then I didn’t have time to see them again during that day. So, they kind of missed out on OT but in the initial stages anyway, when I’m not doing a huge amount because they’re just so heavy, it probably would be beneficial.”– Orthogeriatric Clinical Staff 3*.

Participants identified improvements to the implementation process at the screening stage. To begin with, this intervention may be quite difficult to implement for inpatients who have quite a complex medical background. Thus, by screening the inpatients, additional daily physiotherapy occasions of service can be given to those who will more likely benefit from and be able to participate with the sessions.

*“a screening process for the ones that are slightly higher likelihood of potentially falling, or poor participation, post-operative complications,”– Physiotherapist 5*.

The BOOST intervention involved one session completed by a physiotherapist, which involved mobilisation with the inpatient and any exercises that the physiotherapist deemed appropriate. However, there were some suggestions for a change of structure, including potentially having two mobility sessions and one sit-to-stand session, or just having two longer mobility sessions combined with sit-to-stand and other exercises.

In addition to a change in structure, due to the inclusion of inpatients who may be more complex or frailer than others, some participants believed that a gradual build up to an increased number of exercise sessions may be better.

*“a gradual thing over a couple of days, like one day for the first 24 hours, then you went to two days in 48 hours, then you went three times in sort of the first 60 hours.”– Patient Carer 1*.

There were some frustrations among the alternative workforce about a perceived lack of autonomy with the intervention protocol. Some physiotherapy students felt they could not sufficiently individualise their treatment for the inpatients because they could not progress the exercises without physiotherapist input.

*“I feel like the patient definitely could have done a bit more to tire themselves out. But I just kind of followed the exercise program strictly…” - Physiotherapy Student 3*.

### 5. Future directions of BOOST

Staff found BOOST to be a valuable practice for inpatients and they provided some suggestions for how BOOST could be utilized in the future. There were considerations for implementing BOOST across more sites and examining its effects in a larger study.

*“looking at additional sites…, or whether it’s expanding over a greater period than the 10-week period that we implemented. But I think that was one of the major things, was really looking at the number of patients that we included, is there a way we could have increased how many patients we were able to include.” - Health Service Manager 2*.

Although inpatients and medical staff valued the help from physiotherapy students who were on placement, some proposed that in future, they would benefit from an alternative workforce that consisted of only allied health assistants, as it would save time in having to train and educate students.

*“…with the students, the first couple of weeks… doesn’t free up the physio’s time at all…, because there’s still lots of learning and lots of supervising to be done in the first couple of weeks of their placement. But, if you had an experienced allied health assistant, you wouldn’t have to be providing that kind of assistance.” - Physiotherapist 4*.

## Discussion

This study aimed to describe the experiences of inpatients, carers, physiotherapists, the alternative workforce, orthogeriatric clinical staff, and health service managers on utilising an alternative workforce to administer two additional physiotherapy exercise sessions per day for inpatients after surgery for hip fracture. Five themes emerged from the interviews: (1) individual perceptions of the intervention: inpatients/carer/staff/student, (2) implementation within the service and organisational context, (3) implementation strategies that were effective, (4) improvements to implementation strategies/barriers to implementation/unsuccessful strategies and (5) future directions of BOOST. Overall BOOST was viewed as beneficial and the implementation by the alternative workforce was considered safe, acceptable and was seen to be congruent with the Consolidated Framework for Implementation Research (CFIR) construct. Participants also provided some suggestions to improve the future delivery of BOOST.

Comparisons can be drawn between the themes that we identified and the Theoretical Framework of Acceptability (TFA) which is a seven-component construct [18]. These include: (1) affective attitude, (2) perceived effectiveness, (3) burden, (4) self-efficacy, (5) intervention coherence, (6) opportunity costs and (7) ethicality [18].

Affective attitude was defined as “how an individual feels about taking part in an intervention”, while perceived effectiveness was “the extent to which the intervention is perceived as likely to achieve its purpose” [18]. The BOOST study was able to capture both these notions within all the themes that were discovered, with a mostly positive view. BOOST was perceived as effective, as the additional exercise sessions were viewed by inpatients and the allied health and medical team as being effective for remaining active and providing the necessary and valuable benefits of mobility and strengthening during hospitalisation.

This aligns with the theme that was found by Sims-Gould and colleagues that people with hip fracture valued engaging in physical activity such as walking outside and attending exercise classes or physiotherapy sessions and they believed these were a very important part of their recovery experience and rehabilitation when interviewed one to two years after a fall-related hip fracture [19]. BOOST was also perceived to progress inpatients’ mobility and independence at a faster rate, which participants thought would facilitate care by nursing staff and other allied health staff. These perceptions reflect the findings of a systematic review and synthesis of qualitative data which reported that nurses found assisting with the promotion of independence for patients in hospitals and residents in care homes by collaborating with allied health and medical staff was a priority [20]. BOOST was also perceived to have facilitated earlier discharge of inpatients who were also in a better functional state, which aligns with the quantitative results described by Kimmel and colleagues regarding patient mobility [12]. Thus, BOOST seems an acceptable intervention for elderly inpatients after surgical repair of hip fracture. Implementing the BOOST program for elderly inpatients following a hip fracture may potentially interrupt the vicious cycle of functional decline created by this debilitating injury [4].

The definition of “burden” in the TFA was “the perceived amount of effort that is required to participate in the intervention”, and the component construct of “self-efficacy” was defined as “the participant’s confidence that they can perform the behaviour(s) required to participate in the intervention” [18]. The theme from the present study “implementation strategies that were effective” illustrated these concepts. The orientation of staff to the BOOST intervention was adequate in allowing all participating staff to understand their respective roles and the goals of the study. This may be aligned with the idea of intervention coherence or “the extent to which the participant understands the intervention, and how the intervention works” [18]. However, there were many comments emphasising the importance of having a well-structured orientation process for the alternative workforce in order to reduce the burden. There were suggestions from both physiotherapists and physiotherapy students that the BOOST orientation program should be more interactive and involve practical training. This supports the notion that clinical simulation may be beneficial for physiotherapy students in understanding their roles and for learning [21]. The additional therapy sessions, comprising basic and simple exercises, were able to be implemented in a way that did not impede usual physiotherapy care, provided that the therapists’ and alternative workforce’s daily inpatient lists were up to date.

Timely written communication and handovers are a consideration for inpatient safety and time management. The idea of having a seamless implementation of practice also aligns with the concept of opportunity costs, which has been defined as “the extent to which benefits, profits, or values must be given up to engage in an intervention” [18]. Timely handovers facilitate caseload management by physiotherapists, minimising opportunity costs and inequity of service provision across the hospital. It was also suggested that large caseloads of inpatients with hip fracture may pose difficulty for the implementation of BOOST, although it is uncertain if extra alternative workforce staffing may ameliorate this issue.

It is also unclear whether the level of burden is less in having an alternative workforce that consisted of only allied health assistants, as there were some suggestions from physiotherapists that this would save time in having to train and educate pre-registration physiotherapy students. The pre-registration physiotherapy students who participated in the study reacted positively to the implementation of BOOST. They perceived BOOST to be a novel opportunity to participate in research, as well as beneficial to patients through the provision of evidence-based practice, and it also allowed the students to improve their skills in communication with patients, and time management. Thus, we can see that BOOST was able to be implemented with little to no burden perceived by the pre-registration physiotherapy students.

Similar to the two additional physiotherapy exercise sessions used by Kimmel and colleagues, BOOST appeared to have a low opportunity cost as it incorporated a suitable dosage of treatment and the largely undisrupted scheduling of treatment allowed for participation from all inpatients [12, 18]. It was only suggested by one inpatient that BOOST may be too intensive for inpatients who were in poorer condition. In addition, we did not examine any opportunity cost that may have been perceived by other inpatients or inpatients on other wards.

Often times, as healthcare providers, physiotherapists may be focused on their own management, and may not consider how their treatment plans may impact plans of other allied health professionals. This notion was captured by a clinical staff member who suggested that BOOST may interfere with the time available for their intervention or the interventions of other allied health staff. Perspectives of staff not directly involved with interventions such as BOOST are important to gather to investigate the concept of opportunity costs further in future research [18].

Ethicality is “the extent to which the intervention has good fit with an individual’s value system” [18]. This was demonstrated in the BOOST study through the perception that BOOST was seen as an effective practice for inpatients following a hip fracture as they were able to receive more evidence-based therapy.

We have also used the CFIR in the interpretation of themes that were generated from the interviews, as it not only formed the framework of our semi-structured interview guide, but also serves as a benchmark for implementation research [14]. CFIR integrates 19 models of innovation, dissemination, and implementation, and organises the constructs into five domains: (1) intervention characteristics, (2) outer setting, (3) inner setting, (4) characteristics of individuals, (5) implementation process [14].

As evident in our first theme of “individual perceptions of intervention: inpatients/carer/staff/student”, BOOST was perceived to align with inpatient goals, and was a seen as a viable method of providing higher quality overall care for inpatients with hip fractures. BOOST was viewed as a safe and easy to implement evidence-based practice. Inpatients also saw value in BOOST and appreciated the wide range of exercises available.

The term “outer setting” describes the external factors, such as the organization’s political, social, and economic environment, that have an impact on the way interventions are implemented [14]. This includes factors which are beyond the physiotherapy department and the services offered. In this qualitative study, the interviews generated insights into perceived patient needs. BOOST was perceived to align with clinical standards of hip fracture care and was delivered to a high priority population for the hospitals that were taking part in this study. However, given limited hospital resources and funding for physiotherapists to provide three treatment sessions per day, as well as the impact of COVID-19 drawing resources away from “usual care” and further reducing the workforce availability for hip fracture care, our study aimed to analyse the potential of using an alternative workforce. The BOOST orientation session may need some more practically oriented adjustments to make it more useful for the alternative workforce. Physiotherapy staff perceived that by improving communication within the department and with other health professionals regarding BOOST inpatients would mitigate potential barriers of large caseloads, uncertainty behind inpatient lists, medically complex inpatients who were not suitable for BOOST, and interfering with other therapy times.

“Inner setting” refers to the characteristics of the organisation that will carry out the implementation and encompasses aspects of the structural, political, and cultural contexts in which the process will be executed [14]. BOOST was implemented across two major hospital sites, both of which highlighted equally positive “individual perceptions of intervention: inpatients/carer/staff/student” in regards to the inner setting models of “structural characteristics”, “networks and communications”, “culture” and “implementation climate”. The communication within each of the two physiotherapy departments was also seen as acceptable, although suggestions were made to identify eligible BOOST inpatients earlier in the day and handing over to relevant physiotherapy staff so that larger caseloads are more manageable. The team was able to provide outstanding continuity of care despite this intervention requiring not just a ward-based team effort, but synergy within the entire inpatient team. The inpatients also reported that the physiotherapists and physiotherapy students were able to establish a connection with them, making them feel comfortable and supported while working with a number of different staff members during their hospital stay. BOOST was also perceived to have the ability to improve physiotherapy students’ communications skills, especially around building rapport with their inpatients. Using an alternative workforce in BOOST was seen as a good strategy since it gave physiotherapy student educators more time to meet the needs of students with different abilities. This allowed them the opportunity to concentrate on the students who needed a little more guidance and instruction during their placement, and it also gave the more capable students the opportunity to implement additional therapy sessions for the inpatients with hip fractures.

Most participants were satisfied with the change in implementation via BOOST intervention as it aligned with inpatient goals. In relation to the inner setting category of “compatibility” and “relative priority”, the theme “implementation within the service and organisational context” highlights the perception that BOOST aligned with clinical guidelines for treating hip fractures and was seen as a high priority population for the hospitals that were a part of this study. The “goals” in the category of “goals and feedback” in the inner setting domain were not explicitly discussed in the interviews, however, there was a lot of feedback being communicated back to staff, which was evident in our themes “improvements to implementation strategies/barriers to implementation/unsuccessful strategies”. Some suggestions were made regarding our implementation of BOOST, including comments on the orientation, improving intradepartmental and interdisciplinary communication, and possible changes to the treatment dosage and structure.

The inner setting construct of “learning climate” is perceptible in our theme “implementation within the service and organisational context”. The physiotherapy students found that this project was a valuable learning opportunity, as they took away a variety of clinical and non-clinical skills that they might incorporate into their own practise in the future, which was made possible by including them in the alternative workforce. Additionally, students said that taking part in this study was a great chance to learn more about the research area and that their communication skills, particularly those related to developing rapport with inpatients, had improved. Utilising a different workforce was also thought to be a good strategy because it gave physiotherapy student educators more time to meet the needs of students with different abilities.

Based on the five themes produced from our semi-structured interviews, it is clear that BOOST has “readiness for implementation”, given that it is a model of care that could be easily implemented, is evidence-based, and aligns with patient goals as well as hospital goals and clinical standards of care. There is scope for improvement based on constructive feedback obtained from the interviews and has much potential for upscaling and/or implementation in other areas of care, e.g. cancer care. With regard to the construct of “leadership engagement”, our study included health service managers who provided positive perceptions of the BOOST project and concluded that it was an evidence-based patient-centred model of care. As stated by these health service managers in our theme of “implementation within the service and organisational context”, patients with hip fractures are a priority for hospitals and BOOST is well-aligned with allied health strategy plans and integrates research into practice. The higher frequency treatment certainly assists with exceeding standards of care. Furthermore, the health service managers suggested some “future directions of BOOST” including expansion of the BOOST model of care in terms of size and different sites of implementation such as cancer care and residential aged care facilities.

Inner setting constructs of “available resources” and “access to knowledge and information” can be found within the theme of “implementation strategies that were effective”, as physiotherapists and members of the alternative workforce thought the BOOST study orientation procedure was thorough and that BOOST’s goals and objectives were clear. The extensive selection of exercises allowed for individualisation of care and customization for inpatients who might not have been as compliant on the day due to factors like pain or exhaustion.

In terms of “knowledge and beliefs about the intervention” there are overlaps with the previous three domains of CFIR, as BOOST aligned with hip fracture care clinical guidelines and was an evidence-based intervention model. “Self-efficacy” as well as “individual identification with organisation” was elaborated in our theme of “individual perceptions of intervention: inpatients/carer/staff/student”, where BOOST was thought to be beneficial for staying active and providing the necessary benefits of mobility and strengthening during hospitalisation through an evidence-based approach by inpatients as well as the allied health and medical team.

The constructs of “planning”, “engaging”, “champions”, and “external change agents” were not thoroughly examined through this qualitative study. However, constructs of “opinion leaders” and “formally appointed internal implementation leaders” overlapped with our themes. As previously mentioned in the “inner setting” construct of CFIR, health service managers were involved, and their opinions on BOOST can be found in our theme “implementation within the service and organisational context”. The construct of “executing” has also been explored through the themes where BOOST was perceived as a model of care that could be implemented easily, is evidence-based, matches with hospital aims and clinical standards of care as well as patient goals, and has the potential to be improved by the aforementioned feedback and larger trials.

The main limitation of this qualitative study was that data saturation is unlikely to have been achieved for all groups of participants. Previous work has suggested that 12 or more interviewees are required for data saturation, i.e. when no new themes arise from further interviews [22]. Given the limited number of staff involved in providing care at the two hospital sites, this is an inherent limitation that could only be overcome by conducting a future iteration of the BOOST program across a greater number of hospital sites. We acknowledge that while there was extensive consultation with most relevant healthcare workers in the preparation of the BOOST protocol, not all healthcare groups were consulted (e.g. orthopaedic surgeons) and interviews were not undertaken with the nursing staff providing daily care. Of the 25 inpatients who received the BOOST intervention, 11 inpatients and one nominated carer consented to be interviewed. We deliberately chose to conduct BOOST in two socioeconomically and ethnically disparate areas of Sydney [13]. Hence, although we had good representation of inpatients’ opinions, we may not have achieved data saturation given the diversity. Employing interpreters may have optimised participation from culturally and linguistically diverse inpatients/carers and increased our likelihood of reaching qualitative data saturation, however that was not possible in this study [13]. In addition, although this implementation study has been able to draw from a number of the constructs from CFIR, more explicit and deliberate references to each and every domain may further improve the strength of our intervention. Furthermore, a more diverse range of sites, including regional or rural sites, may potentially offer different perspectives which may add to our understanding of the implementation of BOOST.

Strengths of the study include the fact that inpatients were recruited from two local health districts with diverse sociodemographic profiles which increases the likelihood that the themes generated may be more applicable across a wider spectrum of inpatients. In addition, this is the first study to explore perspectives of inpatients and staff about higher daily frequency of acute exercise rehabilitation following surgical repair of a hip fracture, and as a result, there is a triangulation of data sources from inpatients/carers and staff. This study also triangulated analyses, since the final codes and themes were all agreed upon after discussion between four authors.

Future studies could consider expanding the scope of the intervention by implementing this study across more sites, thus recruiting more participants (inpatients and staff), and also by including higher level managers or executives. The qualitative data presented will influence a larger implementation study and dictate what the intervention may look like, who does it, and potentially what kind of training they might receive.

## Conclusions

In summary, the qualitative data presented here reveal that higher daily frequency of physiotherapy, enabled by utilizing an alternative workforce for two of three daily sessions, after surgical repair of hip fracture, was considered to be safe and was well-received by inpatients, medical and allied health staff, and physiotherapy students. BOOST was perceived to be an effective practice and was successfully implemented in two hospital settings. The results from this qualitative study show that all stakeholders perceived implementations of higher daily frequency exercise as acceptable, in line with goals and beneficial to patients and the health service.

## Data Availability

The datasets generated and/or analysed during the current study are not publicly available as they are considered private health information in our jurisdiction but may be available from the corresponding author on reasonable request.

## Abbreviations

BOOST: Boosting inpatient exercise after hip fracture surgery using an alternative workforce
CFIR: Consolidated Framework for Implementation Research
RCT: Randomised controlled trial
TFA: Theoretical framework of acceptability
